# Ras and Rheb Signaling in Survival and Cell Death

**DOI:** 10.3390/cancers5020639

**Published:** 2013-05-28

**Authors:** Anja Ehrkamp, Christian Herrmann, Raphael Stoll, Rolf Heumann

**Affiliations:** 1 Molecular Neurobiochemistry, Ruhr University of Bochum, 44780 Bochum, Germany; E-Mail: anja.ehrkamp@rub.de; 2 Department of Physical Chemistry1, Protein Interaction, Ruhr University of Bochum, 44780 Bochum, Germany; E-Mail: chr.herrmann@rub.de; 3 Biomolecular NMR, Ruhr University of Bochum, 44780 Bochum, Germany; E-Mail: raphael.stoll@rub.de

**Keywords:** Ras, Rheb, Rassf, mTOR, Tsc, apoptosis, survival, proliferation, tumourigenesis, senescence

## Abstract

One of the most obvious hallmarks of cancer is uncontrolled proliferation of cells partly due to independence of growth factor supply. A major component of mitogenic signaling is Ras, a small GTPase. It was the first identified human protooncogene and is known since more than three decades to promote cellular proliferation and growth. Ras was shown to support growth factor-independent survival during development and to protect from chemical or mechanical lesion-induced neuronal degeneration in postmitotic neurons. In contrast, for specific patho-physiological cases and cellular systems it has been shown that Ras may also promote cell death. Proteins from the Ras association family (Rassf, especially Rassf1 and Rassf5) are tumor suppressors that are activated by Ras-GTP, triggering apoptosis via e.g., activation of mammalian sterile 20-like (MST1) kinase. In contrast to Ras, their expression is suppressed in many types of tumours, which makes Rassf proteins an exciting model for understanding the divergent effects of Ras activity. It seems likely that the outcome of Ras signaling depends on the balance between the activation of its various downstream effectors, thus determining cellular fate towards either proliferation or apoptosis. Ras homologue enriched in brain (Rheb) is a protein from the Ras superfamily that is also known to promote proliferation, growth, and regeneration through the mammalian target of rapamycin (mTor) pathway. However, recent evidences indicate that the Rheb-mTor pathway may switch its function from a pro-growth into a cell death pathway, depending on the cellular situation. In contrast to Ras signaling, for Rheb, the cellular context is likely to modulate the whole Rheb-mTor pathway towards cellular death or survival, respectively.

## 1. Introduction

Small GTPases from the Ras family are well-known molecular switches, that play a pivotal role in the regulation of nearly every cellular process; ranging from cellular growth, differentiation and survival to chemotaxis, transport and apoptosis [1,2,3,4,5,6,7]. Today, the basic mechanisms of their regulation are well understood. GTP loading increases the affinity of Ras proteins to a plethora of downstream effectors, e.g., PI3K or Raf, resulting in the activation of various pathways. GTP hydrolysis to GDP, however, renders Ras in the inactive state. Furthermore, GTP loading is enhanced by guanine nucleotide exchange factors (GEFs), leading to Ras activation whereas GTPase activating proteins (GAPs) accelerate the GTP to GDP hydrolysis, thus promoting Ras protein deactivation [8].

Activation via amino acid substitutions (G12V, Q61L) in all three Ras isoforms (HRas, KRas, NRas) has been extensively shown to promote cellular proliferation, survival and oncogenesis [1,9,10] in various animal models and human tumours [11]. Furthermore, during cell lineage development and in adult systems, activated Ras leads typically via activation of PI3K to survival [7,12,13,14] or via Raf/ERK to differentiation [13,15,16]. Although there is no doubt about these survival-promoting functions, an exciting new image of Ras signaling arose during the last decade; connecting Ras activation with the promotion of apoptosis and other forms of cell death under certain cellular and environmental conditions. Especially the discovery of new Ras effector proteins (Rassfs), mediating the proapoptotic effect of Ras signaling, opened an exciting new field for studying Ras protein family signaling [17,18]. Like Ras, its homologue Rheb is associated with cellular growth, protein biosynthesis, translation and regeneration [19,20], actions mediated mainly via activation of its downstream target mTor [21]. Rheb/mTor signaling is overactivated in many types of cancers, emphasizing their roles as oncogenes [22,23]. However, Rheb signaling is not antiapoptotic *per se*. Dependent on the cellular context, Rheb signaling can enhance cell death [24]. This review seeks to summarize the past and recent findings on HRas and Rheb triggered apoptosis, to contribute to a better understanding of these ambivalently signaling oncogenes.

## 2. Ras Signaling and Induction in Apoptosis: The Pre-2000s

It is known for more than 30 years that the HRas oncogene can stimulate cellular cycling [9,25], differentiation or survival [26,27]. As these are essential processes in multicellular organisms, it may not be surprising that the plane enhancement of these could, according to the cellular situation and system, deregulate the cell. 

Over the last decades, evidences arose that HRas can also induce cellular death under specific conditions, resembling bivalent signaling effects that have been seen for c-myc [28]. With the augmentation of proliferation and the simultaneous induction of apoptosis, an oncogene drives two complementary signals the same time. This is not too surprising if one considers that naturally occurring proliferation comes along with cell death [29,30]. 

### 2.1. Growth Arrest/Senescence

One of the first indications that Ras (from now on, “Ras” is used to denote HRas, if not indicated differently) may not exclusively promote proliferation, was given by Newbold and Overell in 1983. They demonstrated that the expression of oncogenic Ras in fibroblasts results in the induction of growth arrest, phenotypically copying the state of replicative senescence [31].

Similar results were obtained by Serrano and coworkers in 1997 by using primary fibroblasts. Here, the expression of V12Ras lead to G1 arrest, accompanied by an increase of p53. P53 is a potent tumor suppressor that can prevent proliferation via the activation of cyclin dependent kinase inhibitors (CDKIs). Increasing p53 activity induces the accumulation of p21 and p16, both potent CDKIs, thus preventing cycling [32].

### 2.2. Ras in Fas-Mediated Apoptosis

Besides the induction of senescence, Ras activation is implicated in Fas- or TNFα- induced apoptosis [33,34]. Fas receptor activation generally leads to apoptosis [35]. In lymphocytes, however, its activation triggers ceramide synthesis, leading to the activation of Ras and its downstream targets PI3K (that is typically antiapoptotic) and Rac. The activation of Ras is an essential component of FAS-induced apoptosis in this system, as cell death is opposed by the expression of dominant negative (S17N) Ras [33,34]. 

In Drosophila imaginal disc development, Ras activation inhibits apoptosis. On the other hand, the expression of V12Ras promotes both proliferation in postmitotic imaginal tissue and apoptosis of adjacent wildtype cells [36,37]. The finding that expression of oncogenic Ras can either lead to immortalized cells, senescence or apoptosis suggests that the context and system used is crucial to determine the outcome of Ras signaling.

### 2.3. Role of NF-κB

In 1999, Joneson and Bar-Sagi observed that the expression of G12V Ras results in a dosage-dependent induction of apoptosis in both primary (MEFs; mouse embryonic fibroblasts) and secondary cells (fibroblasts). Importantly, Rac (and NF-κB) activation diminished this effect. Rac proteins are involved in NF-κB transcription [38]. Rac is also implicated in the generation of reactive oxygen species (Ros) and NF-κB is activated by Ros [39,40]. At this point it is important to mention that NF-κB also mediates both the induction of apoptosis and survival (among many other functions), depending on cellular type, state and context. Increasing NF-κB activity does not trigger apoptosis *per se*, an additional proapoptotic signal has to be present. However, cerebellar granule cells intoxicated with staurosporine undergo apoptosis while showing increased NF-κB activation. On the other hand, preconditioning of neurons (cerebellar, hippocampal and cortical) with low doses of amyloid beta activates NF-κB, leading to an enhanced neuroprotection [41].

Giving the fact that Rac takes place in the regulation of both transcription and activation of NF-κB and that NF-κB activation can lead to the suppression of apoptosis, it is conceivable that Rac may have at least an indirect protective role against Ras-induced apoptosis [42]. 

Ras is implicated in Rac regulation in two different ways: Firstly, by the activation via PI3Kinase [40,42,43] or, secondly, by interaction with the Rac GEF Tiam [44]. The control of Rac activity, and thus, the impact on cellular Ros levels, might represent an additional way of how Ras affects the overall cellular viability [43,45]. 

The above mentioned findings implicate that not only Ras activity, but also the balance between its various downstream effectors accounts to the cellular outcome. In fact, Ras can induce apoptosis but simultaneously, activation of its downstream target Rac can protect from cell death under certain conditions [45]. This implies that Ras mediated proapoptotic signaling can be compromised by its further downstream prosurvival signals. Nevertheless, it has to be mentioned that Rac activation does not always protect from cell death [44].

### 2.4. Apoptosis via the Erk Pathway

Besides PI3K, one of the best described Ras targets is the extracellular regulated kinase (Erk) [46,47,48]. The activation of Erk1/2 is crucial for the response to a vast number of stimuli, like genomic damage, death receptor activation, nutrient supply or oxidative stress [49,50]. As for Ras, it has also been shown that Erk activation can trigger both growth-supporting and proapoptotic pathways. Although a direct link between Ras activation and proapoptotic Erk signaling is not evident in all studies, the possibility is worth considering and will be discussed in the following. 

Cisplatin is a commonly used anti-cancer drug, but treatment also leads to severe nephrotoxicity. Renal proximal tubal (RPT) cells treated with cisplatin show enhanced Erk1/2 and PKCα activation and undergo apoptosis as a consequence of mitochondrial cytochome c release and caspase3 activation. Interestingly, by blocking Erk1/2 and PKCα signaling, cisplatin induced apoptosis can be partly abolished. Although Erk1/2 and PKCα do not act in the same pathway, their cumulative activation leads to mitochondrial dysfunction and, consequently, apoptosis [51]. Furthermore, the authors report an increase of Erk1/2 activation in RPT cells in response to oxidant injury, followed by apoptotic cell death [52], implying an increasing role of Erk activation in drug mediated renal cell death. Inhibition of Erk has been reported to protect cerebellar granule cells (CGC) from low potassium induced apoptosis [6]. The authors described a protective function of growth-differentiation-factor 15 (GDF15) in several neuronal systems, including CGCs, a well described system to study neuronal degeneration. The observed neuronal survival could be enhanced by pharmacological inhibition of Erk activation, and, consequently, CGN apoptosis was associated with sustained Erk activation. Inhibition of Erk activity lead to an decrease of c-Jun *N*-terminal kinase (JNK) expression and activity, suggesting a possible role of Erk in the regulation of JNK. GDF-15 application and thus, Erk inhibition also resulted in the decrease of intracellular Ros. As Ros can regulate Erk [53,54,55], GDF-15 seems to suppress the generation of Ros, inhibit Erk activity, and thereby causes the attenuation of apoptosis [6].

A study that directly relates Ras-Erk activation to apoptosis was published 2005. The application of calcimycin causes a massive increase of calcium influx, leading to apoptosis in various cellular systems [56,57]. In this study by Li and coworkers, calcimycin-induced intracellular calcium influx activated several MAP kinases prior to apoptosis, including Erk1/2. Interestingly, although calcimycin led to the activation of p38 mitogen activated protein kinase (p38) and also JNK2, only the inhibition of Erk activity via suppression of Ras attenuated the observed cell death. 

### 2.5. Apoptosis via the Intrinsic Pathway

Downstream of Ras/Erk, enhanced calcium influx leads to increased p53 activation, which, in turn, contributes to Bax activation, mitochondrial membrane permeabilisation, cytochome C release, and caspase 3 activation [58].

These consecutive processes represent the classical steps that lead to apoptosis via the *intrinsic* (mitochondrial) apoptotic pathway. The major components regulating this mechanism are proteins that belong to the Bcl-2 (B-cell lymphoma 2) family, consisting of Bcl-2, Bcl-_XL_ and MCL-1 suppressing apoptosis and Bax, Bak and BH3-only proteins, as enhancer of apoptosis [59]. In contrast to the *extrinsic* (receptor-mediated) pathway that is elicited by an extracellular signal, the intrinsic pathway is initiated exclusively inside the cell [60]. 

Bcl-2 is a major component of the intrinsic pathway and plays a crucial role in defending the mitochondrial membrane against permeabilisation. It blocks Ras-induced apoptosis after PKC inhibition, and it was reportet that Ras and Bcl-2 interact under normal, and with even higher affinity under apoptosis stimulating conditions. Furthermore, the presence of activated Ras increases Bcl-2 phosphorylation (and thus, activation), which results in an increase of their association. Consequently, the inhibition of Bcl-2 phosphorylation decreases its affinity to Ras, rendering cells more susceptible to apoptosis [61]. The identification of the subcellular localization of this complex to the mitochondrial membrane gave rise to an attractive explanation of its physiological role: Under basal conditions, Ras resides mainly at cytoplasmic membranes, where it exerts its well-known mitogenic effects. In response to death stimuli, however, it translocates to the mitochondrial membrane, at which interaction with Bcl-2 partly absorbs its proapoptotic signal [62].

There are several reports on direct and indirect crosstalks between Ras and Bcl-2 [63]. For instance, an early study by Davis *et al*. reports constitutively elevated Bcl-2 levels upon Ras-transformation of NIH/3T3 cells, presumably mediated via p53 [64]. In accordance with this, ectopic activation of Raf in breast cancer (MCF-7) cells yields an increase in Bcl-2 protein levels [65]. Other studies show both enhanced Ras and Bcl-2 protein expressions [66] or elevated amounts of Bcl-2 mRNA [67] in cancer cells upon drug treatment. Nevertheless, it remains controversial if Ras and Bcl-2 truly interact under physiological conditions or in other cell lines than Jurkat cells, as described by Chen and Faller. In addition to a regulation of the intrinsic apoptotic pathway, other data show that Ras is involved in the regulation of expression of proteins involved in it: for example, Ras influences the expression of p53, Bax, Bnip3 and Bcl-2 [58,61,68,69]. However, the understanding of the exact mechanism(s) how Ras promotes apoptosis via the intrinsic pathway remains enigmatic.

### 2.6. Apoptosis via the Extrinsic Pathway

Besides regulating apoptosis via the intrinsic pathway, Ras/Erk signaling might also be involved in triggering apoptosis via the extrinsic pathway. Phosphoprotein enriched in astrocytes (PEA-15) is implicated in the coordination of both apoptotic and non-apoptotic pathways by binding to FADD, procaspase8 or e.g., Erk, thus changing their intracellular localization. Overexpression of PEA-15 blocks Erk-dependent transcription and, hence, PEA-15 expression levels control the biological outcome of the Erk pathway [70]. The connection between Erk1/2 and FADD/procaspase8 via the interaction with PEA-15 might explain its dual role between survival and apoptosis. 

Additional indication that Ras/Erk modulates the extrinsic pathway was provided by the finding that the expression of HRas (and also KRas) in Caco2 cells leads to an upregulation of Trail (TNF related apoptosis inducing ligand) receptors. Thus, the elevated amount of death receptors in cells expressing oncogenic Ras makes these cells more vulnerable to (TRAIL induced) apoptosis. Considering that nearly 50% of all human colon cancers carry activating mutations in any of the Ras genes, treating patients with TRAIL agonists might potentially allow the specific destruction of transformed cells [71].

## 3. Ras Signaling and Induction in Apoptosis: The Recent Findings

The recent findings with the identification of Nore1 (Rassf5) in 1998, a new area of investigations on proapoptotic Ras signaling emerged. Nore1 was identified as novel binding partner of activated (G12V) Ras in a yeast two hybrid screen [17]. The protein exists in multiple isoforms, generated either by different splicing or different promoter usage. Among these, the most prominent ones are Nore1a and Nore1b, which possess different roles in cellular regulation. Nore1a regulates cellular motility, cell cycle control, apoptosis, and stability of microtubules, whereas Nore1b is rather implicated in adaptive immune defense [72]. In this review, the term “Nore” will be used to denote all isoforms, if not indicated otherwise.

Remarkably, Nore1a reveals an ubiquitous expression pattern in non-transformed cells, whereas its mRNA is almost completely downregulated in many cancers due to promoter methylation. The investigation of downregulation of Nore1b transcripts in transformed cells did not reveal any promoter methylation, which indicates a possible role of Nore1a as tumor suppressor [18,73]. 

Nore binds to Ras directly via its Ras association (RA) domain *in vivo* and *in vitro*, and this interaction strongly depends on GTP bound state of Ras [17,74,75]. Follow up studies revealed that Nore binds to endogenous Ras after serum stimulation. Additionally, using yeast-2-hybrid assays, interactions with other GTPases like Rap 1/2, RRas 1/2 and MRas were observed, showing similar affinities [74]. 

### 3.1. A trimeric Complex of Ras, Nore and MST1

Interestingly, Nore additionally interacts with the proapoptotic kinase MST1 (mammalian sterile20-like kinase). The overexpression of G12VE37G HRas (activated but unable to bind Raf or PI3K) or G12V KRas results in the induction of apoptosis via recruitment of MST1-Nore1. However, this effect was not observed for the overexpression of the G12V HRas single mutant. The observation that G12VE37G but not G12V HRas induces apoptosis could be rationalized by the lack of activation of antiapoptotic pathways via Raf/PI3K by the double mutant, whilst still being able to interact with Nore1a. The finding that the interaction between Ras-Nore-MST1 occurs also under physiological conditions [76] triggered a more intense investigation on the regulation of this complex Nore1a inactivation enhances the resistance of murine liver cells to TNFα-induced apoptosis [77] However, it remains unclear, whether Nore1a overexpression induces apoptosis in mammalian cells *per se* [76].

Although Nore1a binding generally inhibits MST1 activity, the addition of GTP-Ras to this complex results in an increase of MST1 activity. Thus, only MST1 molecules still complexed by Nore1a and Ras-GTP undergo persistent activation due to translocalisation to the membrane, suggesting rapid inactivation of non Ras-GTP bound MST1 molecules by endogenous phosphatases [78]. A compelling model, how these findings could be explained arose from the observation that the recruitment to the plasma membrane also multiplies MST1 activity [76].

Under basal conditions, Nore-MST1 complexes constitute quickly accessible pools restrained within the cell. Upon apoptotic stimuli, however, Nore1a binds Ras-GTP, which localizes Nore1a-MST1 to the plasma membrane. Ras-mediated recruitment of MST1-Nore1a leads to an increase of MST1 Thr^183^-phosphorylation and thus, activation of MST1. Thus, Nore1a might not only constitute an inactivating binding partner for MST1. It also seems to act as scaffolding protein to connect MST1 with activator and substrate molecules, respectively, which results in an increased activity of MST1 [77,78].

### 3.2. Nore1a in Growth Suppression

As mentioned before, Nore1a is a tumor suppressor that induces growth inhibition and cell cycle arrest in a variety of cancers and cell lines investigated. Although the exact mechanism, how these growth inhibitory functions are achieved remains elusive, Nore1a localization to microtubules and centrosomes seems to be crucial [79,80,81].

There are conflicting reports on the role of Ras in Nore mediated growth inhibition: while Vos and coworkers found that it is dependent on Ras, Aoyama and coworkers reported that it is independent of an interaction between Nore and Ras, or even the presence of Ras. However, Ras has been implicated in growth suppression in other contexts (e.g., senescence) before. Taken together, the involvement of Ras in Nore-mediated growth inhibition is only speculative [32,82,83].

Indications for this hypothesis came from Vos and Coworkers in 2003: They demonstrated that Nore-induced growth inhibition and, subsequently, originated apoptosis, is enhanced by activated and attenuated by dominant negative Ras [79]. This was supported by Moshnikova and coworkers in 2006. The minimal region of Nore that is responsible for its growth inhibitory effects (aa191–361) corresponds to the Ras association (RA) domain. This region mediates binding to microtubules, thus affecting Nore1a subcellular localization. Furthermore, it conveys suppression of the Erk pathway by a yet unknown mechanism. In addition to binding to active Ras, Nore has to associate with the cytoskeleton in order to accomplish growth suppression. However, besides the presence of activated Ras, another, yet unknown stimulus has to be present to finally convey it [84]. An attractive explanation for Nore-mediated growth inhibition came from Bee and coworkers in 2008. They showed that Ras and tubulin competitively bind to Nore1 RA domain. Binding of Nore to tubulin induces microtubule polymerisation (growth/stabilisation), whereas binding to Ras suppresses this effect. Moreover, the binding of Ras to Nore in competition to other Ras effectors could explain suppression of the Erk pathway; Nore RA overexpression leads to sequestration of Ras, which, in turn is not capable of inducing Erk activation anymore [85]. 

### 3.3. Rassf1

Rassf1 is another well characterized member of Rassf. Like Nore, it is capable of binding GTP-H/KRas *in vitro* and also in an endogenous environment [86,87,88,89]. Rassf1 exists in various splice variants and the major isoforms, Rassf1a and Rassf1c are widely expressed in normal tissues [18]. However, their loss of expression due to promoter methylation is one of the most common events in a wide variety of human cancers [90], which suggests a role as tumor suppressor genes [91]. Consequently, re-expression of Rassf1a in tumor cell lines results in a decrease of cell viability, growth, invasiveness and substrate independence [92,93,94,95,96]. 

The exact role of Rassf1c is not yet understood. While some authors found no change in cellular growth or apoptosis [93,97,98], others observed an increase of proliferation or apoptosis subsequently to Rassf1c overexpression [86,99]. Interestingly, Michele Vos and coworkers discovered a strong influence of Rassf1c on Ras-mediated apoptosis, as activated Ras considerably increased, and dominant negative Ras diminished Rassf1c-mediated cell death. A more detailed study investigated the implication of Rassf1c in the transmission of proapoptotic signals after DNA damage. Genotoxic insults, such as UV radiation, lead (among others) to the activation of JNK. It was not known, however, what transmits the nuclear signal to the cytoplasm, where JNK activation occurs. In the nucleus, Rassf1c is associated with Death domain associated protein (Daxx), a proapoptotic protein that is degradated upon cellular damage. After Daxx degradation, Rassf1c is released from the nucleus, complexes with Ras and translocates to the cytoplasic microtubules where it to activates JNK. However, why Rassf1c associates with Ras at the membrane, instead of activating JNK in the cytoplasm immediately, remains elusive. Furthermore, whether a direct interaction with Ras is necessary for Rassf1c transportation, or whether this interaction is indirect via Nore remains elusive at this stage [74,100].

Like Nore, Rassf1a interacts with MST1. Furthermore, Ras-induced apoptosis is inhibited by the disruption of MST1-Rassf1a/Nore1a complexation. Giving the fact that excessive Ras signaling induces oncogenic transformation, Rassf1 dependent recruitment of MST1, thus, promoting cell death after extensive Ras-signaling might act as a protective mechanism against transformation [76,78,101]. 

Besides MST1, Rassf1a interacts with modulator of apoptosis 1 (MOAP1), a proapoptotic kinase known to bind to Bax, thus modulating its activity. This interaction is stimulated by the presence of G12V *K*Ras, resulting in an enhancement of cell death. Unfortunately, the authors do not mention if *H*Ras is implicated in the modulation of Rassf1a-MOAP complexation [87]. 

## 4. Rheb Signaling and Induction of Apoptosis

Ras homologue enriched in brain (Rheb) is a small GTPase from the Ras family of monomeric G-proteins that is highly related to Ras. Due to the sequence and structural similarity, similar functions to those of Ras were immediately assumed after its discovery in 1994 [102,103]. Like Ras, Rheb is implicated in a vast amount of cellular processes like cellular growth, protein translation, cellular differentiation, protein biosynthesis and autophagy [19,20,22,104,105]. Rheb activity is controlled by its GAP Tsc1/2, which, when inactivated, cause tuberous sclerosis. GTP-bound Rheb activates Tor (target of rapamycin) kinase that is conserved from Drosophila (dTor) to mammals (mTor) and is strongly inhibited by rapamycin. Tor exists in two multiprotein complexes (mTorc1/2), containing among others Raptor, mLST8, mTor, to form a rapamycin sensitive, or Rictor, Sin1 and Protor, to yield a rather, but not completely rapamycin insensitive complex. However, the functions and signaling of mTorc2 are still much less understood than those of mTorc1 [19,106]. As the growth promoting roles of the Rheb-Tor pathway are well characterised (excellent reviews by [19,69]), it is not surprising that its deregulation can have devastating effects on a cellular system. Here we will focus on the de-regulations of Rheb and its direct upstream and downstream effectors, Tsc1/2 and Tor that can ultimately lead to apoptosis.

### 4.1. Rheb Transcription and Posttranslational Modifications

Initial studies showed that Rheb mRNA is upregulated after seizures or NMDA receptor activity in hippocampal neurons like an immediate early response gene [102]. Further, UV irradiation has a similar effect on Rheb mRNA levels and implies that Rheb is involved in sensitizing cells to death after proapoptotic stimuli [107]. Further studies revealed that Rheb expression in the CNS is upregulated after injury, both on the protein [108] or mRNA levels [109]. The studies suggested that Rheb activity may not only be regulated by its GTP-loading state but also by its level of expression. Probably, expression levels may be a better indication for Rheb activity, as Rheb, compared to Ras, already shows a high activation state even under basal conditions [110]. Additionally, Rheb activity is commonly regulated via farnesylation of its *C*-terminal CAAX motiv, thus leading to membrane localisation and an increase of signaling activity [102]. More recently it became evident that Rheb activity is also regulated by phosphorylation [111] or redox regulation [112]. All together this promotes the idea that the regulation of Rheb activity is not only controlled by cellular GTP/GDP levels, but that a major mechanism additionally consists of posttranslational modifications.

### 4.2. The Two Signaling Faces of Rheb

However, it was not clear as to whether Rheb promotes growth and regeneration or cell death. Recently, Karassek and coworkers showed that transient overexpression of Rheb in HeLa cells followed by UV irradiation, TNF alpha or tunicamycin intoxication led to a mTorc1 dependent enhancement of apoptosis. This effect is also apparent after excitotoxic stimulation of cortical murine neurons with glutamate. Knockdown of endogenous Rheb reversed the enhanced apoptotic effect, which suggests that the enhancement of apoptosis in HeLa cells or neurons, respectively, occurs in a Rheb dependent manner. Overexpression of Rheb without a proapoptotic stimulus does not lead to an enhanced apoptosis. In order to investigate possible structural implications of the Rheb mediated apoptotic enhancement, NMR spectroscopic analysis were carried out. The results showed that Rheb was able to bind to the Ras binding domain of Raf (Raf-RBD) by a specific pattern similar to Ras, yet by 1,000-fold lower affinity, thus excluding a Ras like signaling pathway by Rheb. The novel proapoptotic signaling by Rheb was mediated by apoptosis signal regulating kinase-1 (ASK-1) [24]. 

In concordance with this, Zhou and coworkers found that enhanced Rheb activity inhibits the formation of aggresomes and leads to the sensitization to apoptosis in response to misfolded protein stress. However, this effect was not dependent on mTorc1. Most strikingly, Rheb disrupts aggresome formation by intercepting the dynein dependent transport of ubiquitinated cargos destined for degradation [105]. In 2010, Ma and coworkers published a study showing that Rheb regulates the interaction between FKBP38 (an inhibitor of mTOR) and the antiapoptotic protein Bcl-2. While bound to GTP, Rheb binds FKBP38 and thus prevents its association with mTorc1, enabling mTorc1 to be active [113]. FKBP38 interacts with the antiapoptotic proteins Bcl-22 and Bcl_XL_ and it has been shown to participate in targeting these to the mitochondria. There, Bcl-22 and Bcl_XL_ exert their antiapoptotic effects by binding Bak and Bax, thus preventing permeabilisation of the mitochondrial membrane [114,115,116,117]. However, in the same study, Ma and coworkers found that Rheb regulates both the interaction between, and the release of FKBP38 and Bcl-22/Bcl_Xl_. Strikingly, this interaction is dependent of the energy level of a cell, but not rapamycin-sensitive, suggesting an mTOR independent mechanism. They propose a model of how Rheb can either regulate the antiapoptotic cellular program (while active) or of how it may facilitate the induction of apoptosis under suboptimal conditions. Thus, when the cells are starved and Rheb is bound to GDP, Bcl_Xl_ and Bcl-2 are still recruited to the mitochondria, but Rheb is unable to release them from FKBP38, which ultimately disables them to bind and inhibit Bax and Bad [113]. This provides an elegant model of how the switch between pro- and antiapoptotic signaling mediated by a single protein can be achieved on the cellular level. Taken together, not only canonical pathways play a role in Rheb-mediated apoptosis, but also non-canonical pathways that are not dependent on mTOR.

Another example of how Rheb regulates divergent outcomes within the same context was given by Honjoh *et al.* in 2009 [118]. *C. elegans* is a commonly used model to study mechanisms of aging and it has been shown that caloric restriction or intermitted fasting can both increase lifespan and decrease the occurrence of age-related disorders. In worms, Rheb seems to be implicated in the regulation of life span in two different ways. Firstly, knockdown of Rheb (but not Tor) plus unlimited food supply or caloric restriction of control animals, respectively, extended their lifespan, thus suggesting an antagonizing role of Rheb in longevity. Secondly, the combination of caloric restriction plus knockdown of Rheb or Tor versus caloric restriction in control animals suppressed longevity. These results indicate both Tor dependent and independent pathways for Rheb-mediated control of *C. elegans* lifespan.

### 4.3. Rheb in the Regulation of Autophagy

Autophagy is a crucial cellular pathway that is implicated in cellular survival. This process is generally described as “cellular self-digestion”, to protect the organism as a whole from damage of one single cell, or to generate nutrients from cells’ contents in case of energy starvation, thus, protecting a cell from death. The regulation of autophagy is tightly coupled to the regulation of apoptosis and *vice versa* [119,120]. 

It has been known for a long time that Rheb/mTor negatively regulates autophagy in various organisms [121]. However, recent studies indicate that Rheb activation might also induce cellular damage through the inhibition of autophagy. The suppression of autophagy can have devastating effects: In the brain, it causes neurodegenerative phenotypes, including neuronal death, as result of cytoplasmic accumulation of insoluble aggregates [122,123,124]. Cancer is the first disease that was genetically linked to a dysfunction of autophagy, [120] and autophagy is suppressed in many human tumours, while deregulated Rheb signaling is also appearent in many types of tumors [22,125,126,127]. More specifically, genes that inhibit autophagy, like mTor and Rheb, are upregulated in many tumours, whereas autophagy-promoting genes. like Tsc1/2 and Pten, are downregulated [120].

It is not yet entirely understood how the Rheb-mTor pathway exactly suppresses autophagy, but it is clear that these proteins play a crucial role. However, it is evident that stimulation of autophagy does not have an unambiguous effect in the treatment of cancer, as autophagy can be both protective and harmful for cancer cells [120,128]. Thus, although it appears to be an attractive hypothesis, the connection between deregulated Rheb/mTor signaling, suppression of autophagy, and the development of tumours requires further investigation in depth.

### 4.4. Tor Kinase

Downstream of Rheb, the serine/theronine kinase mTor signals via two different multiprotein complexes, regulating several crucial cellular functions (see above). Although mTor and the mTor pathway are activated in a wide variety of human cancers, being implicated in tumourigenesis [126,129,130,131], an emerging body of evidence puts the output of mTor signaling in a more context-dependent image. Chen *et al.* showed, that cadmium (Cd^2+^) exposure to neuronal cells activates mTor signaling, which leads to apoptosis. However, treatment with rapamycin prevented Cd-induced apoptosis, showing the importance of mTor activation in this context. Cd-induced apoptosis was assigned to be due to Ros production, eventually increasing mTor activity [132]. In 2005, Kim and coworkers found a reduction of basal Ros levels in transformed haematopoietic cells by the inhibition of PI3K or mTor [133], however, without elucidating the exact molecular mechanism. Ros have been previously described to trigger mTor activation and consequently, the induction of apoptosis [133,134]. However, elevation of cellular Ros levels may contribute to the pathogenesis of many cancers [135,136]. These discoveries opened a yet unknown field on mTor signaling in via Ros in transformation and apoptosis.

Feeding of rapamycin to aged (female/male) mice prolongs their lifespan up to 14/9%, respectively. The delay of death in living animals seems to be due to the postponed development of cancer and mechanisms of aging [137]. Very recently, Iglesias-Bartolome and coworkers found that mTor inhibition by rapamycin prevents senescence of epithelial stem cells. More specifically, the inhibition of mTor leads to a decrease of Ros, as well as DNA damage, in cells stressed by UV light [138]. A decrease of Ros production after mTor inhibition had been observed before, as reported by Kim and coworkers in 2005 (see above) and others [139,140]. However, the underlying mechanism remains elusive. Iglesias-Bartolome and coworkers reported that this is mediated by elevated mitochondrial superoxide dismutase (MnSod) activity, as the decrease of Ros and MnSod are correlated. MnSod defends cells against mitochondrial oxidative damage via the detoxifation of superoxide [141]. Thus, the enhanced expression of MnSod after rapamycin treatment presumably leads to a reduction of Ros, oxidative stress and, ultimately, senescence. However, as the application of rapamycin does not enhance MnSod RNA levels in epithelial stem cells, the exact mechanism of how mTor activity and MnSod expression are coupled remains elusive [138].

### 4.5. Tsc1/2 Complex

It is known for long that loss of function mutations in either or both of Tsc1/2 proteins cause seizures, cognitive defects and benign tumors in multiple tissues, yielding to a disease known as tuberous sclerosis [142,143]. The Tsc1/2 proteins, also known as hamartin/tuberin, form a heterodimer acting as GAP for Rheb, thus inhibiting protein translation, biogenesis, and translation or cell growth by inhibiting mTor activity [144,145]. Thus, deregulation of Tsc gene products (either by genetic events or signaling deficiencies) directly causes deregulation of Rheb and mTor.

Elevated Tsc signaling, e.g., by nutrient starvation, decreases the phosphorylation of the most common targets of mTor, *i.e.*, ribosomal S6 kinase and eukaryotic initiation factor 4E binding protein-1 (4EBP1), thus suppressing protein translation and cellular growth [146,147,148]. In contrast, Tsc inhibition enhances S6K and 4EPB1 phosphorylation [149,150,151,152]. This regulatory mechanism protects cells from “wasting” crucial nutrients on protein biogenesis under starving conditions [148]. Consequently, a deregulation of Tsc signaling results in severe disturbances of the cellular energy metabolism leading, to fatal consequences.

Uncontrolled protein synthesis and/or nutrient deprivation can cause unfolded protein stress (Upr) [153]. In 2008, Ozcan and coworkers demonstrated that loss of Tsc1 or Tsc2 genes causes ER stress and unfolded protein response in tumours and MEFs, which, in turn facilitates apoptosis [154]. This was confirmed in Tsc2-deficient primary hippocampal neurons that showed an enhanced basal- and ER-stress induced cell death in response to hyperactive mTor. In addition, consistent with the well-known coupling between ER and oxidative stress responses [155] ER stress resulted in an increase of cellular Ros-levels in hippocampal neurons [156]. In accordance with the previous results, Kang and coworkers observed a higher vulnerability of both Tsc1^−/−^ and Tsc2^−/−^ mutant MEFs to ER stress, compared to their wildtype counterparts. Consistently, this effect could be reversed by the reconstitution of Tsc. Interestingly, only knockdown of Rheb or Raptor, an essential component of mTorc1, but not treatment with Rapamycin, attenuated the observed susceptibility, which indicates a partial mTorc independence and, thus, the involvement of a yet unknown regulatory component [145]. The studies showing that the deregulation of Tsc may sensitize cells to death support the view that upregulation of the Rheb/mTor pathway mediates proapoptotic effects under specific conditions. Unfortunately, it has not been investigated whether proapoptotic Rheb/mTOR pathways might be coupled to those that are Ras-dependent. It will be exciting to see how these new observations may lead to a better understanding of stress response in cancer cells, enabling a selectively targeted therapy to diminish the side effects on healthy tissue.

## 5. Conclusions

**Figure 1:**
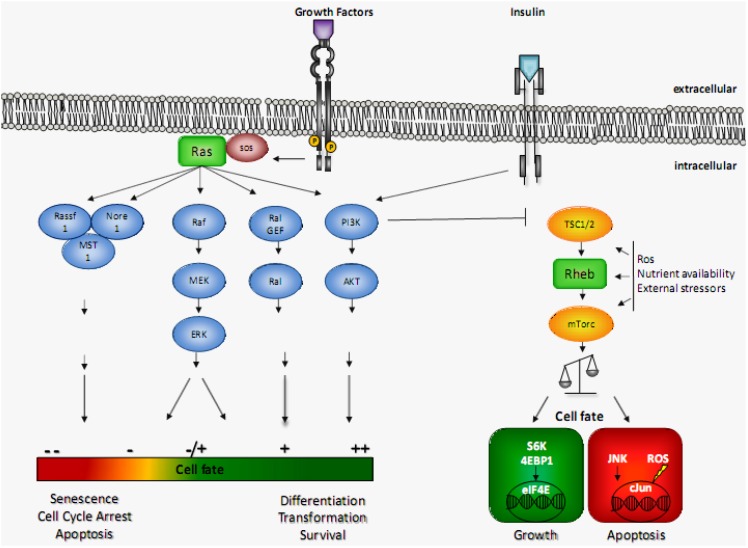
Pro- and antiapoptotic Ras and Rheb signaling based on different effectors and cellular context. Growth factors and insulin lead to the activation of Ras or Rheb GTPase, via their specific receptors. Downstream of activated Ras, a pleiotropy of downstream targets (*i.e.*, PI3K, Raf. Ral Gef, Rassf1/5) are activated that, cooperatively, contribute to the overall cellular signaling outcome. Activated Rheb, however, stimulates the mTor pathway, leading to either pro- or antiapoptotic signaling, which critically depends on the cellular context. For details see text.

Increased activation in both Ras or Rheb signaling pathways are found in wide variety of human tumours, including bladder, brain, kidney, skin, and many other tissues [11,126]. However, during the last years, the view that these GTPases are pure oncogenes has been challenged. An increasing number of studies show that Ras and Rheb signaling is more complex than just enhancing mitosis, differentiation and survival or cellular growth, respectively. In fact, both proteins may play a major role in cellular degeneration. The observation that pathways regulating cellular survival are tightly coupled to those regulating cellular death is widely assumed to be a protective mechanism against transformation [86]. The broad effects mediated by GTP-bound Ras or Rheb reflect, on the one hand, the huge array of effector proteins and pathways that are influenced by their activation state [157,158]. On the other hand, the specific subcellular localization, post-translational modifications or even the cellular context give rise to a specific signaling readout [159]. Overall, it seems likely that the decision as to whether Ras signaling pursues a pro-growth or a pro-apoptotic outcome depends on the balance between the selectively activated or deactivated downstream pathways, whereas for Rheb/mTor this decision appears to be determined by integrating the overall cellular context (Figure 1). It will be fascinating to explore how Ras and Rheb might counteract or complement each other in a given context. We observed that Ras attenuates Rheb-enhanced apoptosis in HeLa-cells [24]. Nevertheless, more work is needed in this field of research until we will conclusively understand the complex signaling of these potentially death promoting oncogenes.
